# Integrated Multi-Omics Reveals Disruption of Lipid–Steroid Regulatory Axis Underlying Chronic Heat Stress-Induced Ovarian Dysfunction in Mice

**DOI:** 10.3390/ijms27198671

**Published:** 2026-09-28

**Authors:** Farwa Nazir, Muhammad Ammar Baibrus, Muhammad Tariq, Mohamed Tharwat, Shiyuan Pei, Yang Wen, Jie Shen, Meilian Su, Qiuxia Liang, Guangbin Zhou, Fahad A. Alshanbari, Guozhi Yu

**Affiliations:** 1 College of Life Sciences, Sichuan Agricultural University, Xinkang Road 46, Ya’an 625014, China; farwahunjra420@gmail.com (F.N.); ammarbaibrus0@gmail.com (M.A.B.); 413382580psy@gmail.com (S.P.); winy0101@163.com (Y.W.); shenjieee@outlook.com (J.S.); dxgjbftjb15528965@163.com (M.S.); 2022316058@stu.sicau.edu.cn (Q.L.); 2 College of Animal Science and Technology, Nanjing Agricultural University, Nanjing 210095, China; tariq@stu.njau.edu.cn; 3 Department of Clinical Sciences, College of Veterinary Medicine, Qassim University, P.O. Box 6622, Buraidah 51452, Saudi Arabia; atieh@qu.edu.sa; 4 Institute of Animal Genetics and Breeding, College of Animal Science and Technology, Sichuan Agricultural University (Chengdu Campus), Wenjiang, Chengdu 611130, China; zguangbin@sicau.edu.cn; 5 Department of Medical Biosciences, College of Veterinary Medicine, Qassim University, P.O. Box 6622, Buraidah 51452, Saudi Arabia

**Keywords:** heat stress, ovarian dysfunction, multi-omics integration, lipid metabolism, steroidogenesis, PPAR signaling, histopathology

## Abstract

Heat stress is an escalating environmental challenge that adversely affects mammalian reproductive physiology, yet the integrated molecular mechanisms through which ovarian tissue responds to sustained heat stress remain incompletely understood. This study investigated the effects of chronic heat stress on mouse ovarian tissue using an integrated multi-omics approach. Female C57BL/6 mice were exposed to heat stress at 42 °C for 20 min daily over 30 days. Whole ovarian tissues were subsequently analyzed using RNA sequencing, DIA-based proteomics, untargeted metabolomics, histological examination, and RT-qPCR validation. Histological analysis showed a significant reduction in secondary follicle profiles in heat-stressed mice, whereas primary and antral follicle profiles showed inter-animal variability and did not differ significantly between groups. Transcriptomic and proteomic analyses identified altered expression of lipid metabolism- and steroidogenesis-related molecules, including *Apoa1*, *Apoa2*, *Lpl*, *Fads2*, and *Hsd17b2*. Metabolomic profiling further showed marked changes in metabolites related to lipid metabolism and steroid hormone biosynthesis. Integrated pathway analysis revealed recurrent enrichment of pathways associated with lipid metabolism and endocrine regulation, particularly PPAR signaling, cholesterol metabolism, and steroid hormone biosynthesis. RT-qPCR validation confirmed altered expression of selected genes involved in lipid transport and steroidogenic pathways. These findings suggest that chronic heat stress may disrupt ovarian metabolic and endocrine homeostasis through alterations in lipid–steroid regulatory pathways. This study provides systems-level evidence supporting the involvement of interconnected metabolic pathways in heat stress-associated ovarian dysfunction.

## 1. Introduction

Chronic heat stress has become an important environmental concern affecting reproductive health in humans and animals, particularly as climate change increases the frequency and severity of extreme temperature events [1,2]. High environmental temperatures can trigger systemic and cellular stress responses and disturb endocrine balance, metabolic homeostasis, and cellular function [3]. The ovary is highly sensitive to thermal stress because follicular development, oocyte maturation, and hormone production depend on coordinated cellular interactions and tightly regulated metabolic processes [4,5]. Experimental and epidemiological studies showed that exposure to high temperatures affected follicular development, reduced oocyte quality, disrupted estrous or menstrual cyclicity, and, as a consequence, lower fertility [6,7]. These adverse effects have been widely reported in experimental animals and livestock species exposed to heat stress, highlighting the biological relevance of thermal stress-induced reproductive dysfunction [8,9].

Increasing evidence indicates that heat stress can alter multiple cellular and physiological pathways involved in ovarian function [10]. Experimental studies have shown that thermal stress can induce oxidative stress, mitochondrial dysfunction, and apoptotic signaling in ovarian cells, contributing to impaired follicular development and reduced oocyte quality [11,12]. Heat stress was also shown to affect steroidogenic enzyme activity, disturb cholesterol utilization, and suppress reproductive hormone production in ovarian tissue [13,14]. Collectively, these findings suggest that thermal stress can disrupt both the structural integrity and endocrine function of the ovary.

The ovarian follicle is the basic functional unit of the female reproductive system and depends on metabolic communication among oocytes, granulosa cells, and theca cells to maintain follicular development and endocrine function [15,16]. Lipid metabolism and steroid hormone biosynthesis are closely interconnected processes that are essential for ovarian function [17,18]. Lipids serve as membrane components, energy sources for rapidly proliferating follicular cells, and precursors of signaling molecules involved in follicular development [19,20]. Steroid hormones, particularly estrogen and progesterone, regulate follicular maturation, ovulation, and reproductive cyclicity [21]. Cholesterol is the universal precursor of steroid hormones and must be efficiently transported, stored, and metabolized within ovarian cells through lipoprotein-mediated and intracellular lipid-processing pathways [22]. Therefore, lipid metabolism, cholesterol transport, and steroidogenesis form an interconnected metabolic network that supports ovarian endocrine activity [23,24]. Disruption of this lipid–steroid homeostasis may have important consequences for follicular function and development and reproductive potential [25].

Previous omics-based studies have revealed that heat stress and other physiological stress conditions induce coordinated molecular alterations involving stress-response mechanisms, metabolic regulation, and endocrine-related pathways across biological systems [26,27]. Because gene expression, protein abundance, and metabolite production are highly interconnected processes, single-omics analyses may not fully explain the coordinated molecular responses underlying ovarian dysfunction during chronic heat stress [28,29]. In particular, integrated multi-omics investigations examining the relationships among lipid metabolism, cholesterol regulation, and steroid hormone biosynthesis in heat-stressed ovarian tissue remain limited. Therefore, the present study applied an integrated multi-omics approach combining transcriptomic, proteomic, and untargeted metabolomic analyses to investigate molecular alterations associated with chronic heat stress-induced ovarian dysfunction in mice. Histological analysis and RT-qPCR validation were further performed to support the omics findings. We hypothesized that chronic heat stress may alter interconnected pathways involved in lipid metabolism, cholesterol transport, and steroid hormone biosynthesis, thereby contributing to ovarian dysfunction. This study aimed to identify convergent molecular pathways potentially involved in chronic heat stress-associated ovarian injury.

## 2. Results

### 2.1. Histological Analysis by Hematoxylin and Eosin Staining

Hematoxylin and eosin (H&E) staining revealed follicles at different developmental stages in ovarian sections from both control (CK) and heat-stressed (HS) groups (Figure 1). Complete ovarian cross-sections and corresponding higher-magnification images were used to illustrate the presence of primary, secondary, and antral follicles. The complete ovarian cross-sections shown at low magnification (200 µm scale bar) correspond to the sections used for follicle-profile quantification. The higher-magnification images were provided only to illustrate follicle morphology and classification criteria. The overall ovarian morphology appeared preserved in both groups, with identifiable follicles at different developmental stages. Quantitative histological analysis was performed using four CK and four HS mice (*n* = 4 biological replicates per group). Both ovaries from each mouse were examined, and follicle counts from Ovary 1 and Ovary 2 were averaged to generate one animal-level value for each follicular category. Individual biological values and group means ± SD are presented in Figure 1, while detailed bilateral follicle-count data are provided in Supplementary Table S8. The number of primary follicle profiles showed no apparent difference between CK and HS groups. In contrast, secondary follicle profiles were reduced in the HS group compared with the CK group. Antral follicle profiles showed inter-animal variation but remained comparable between groups. Together, these findings indicate that repeated heat stress primarily affected the distribution of developing follicles, with a notable reduction in secondary follicle profiles, whereas primary and antral follicle profiles were relatively maintained. These morphological observations provide tissue-level evidence supporting subsequent molecular analyses.

### 2.2. Transcriptomic Alterations in Ovarian Tissue After Chronic Heat Stress

Transcriptomic profiling was performed on ovarian tissues from control (CK) and heat-stressed (HS) mice following 30 days of chronic heat stress at 42 °C for 20 min daily, yielding a total of 41,962 genes in the raw data. After filtering, 11,231 genes were retained for further analysis. Genes with extremely low read counts across samples were excluded during preprocessing and normalization to improve the reliability of downstream differential expression analysis. Principal component analysis (PCA) revealed clear separation between the control and heat-stressed groups, with PC1 explaining 34.1% and PC2 explaining 24.7% of the total variance, indicating a significant transcriptional shift associated with chronic heat stress (Figure 2A). Differential expression analysis, using a fold change threshold of |FC| ≥ 1.5 and adjusted *p*-value < 0.05, identified 67 upregulated and 104 downregulated genes, while 11,059 genes showed no significant change (Figure 2B). The full list of differentially expressed genes (DEGs), including the corresponding fold change and *p*-values, is provided in Supplementary Table S1 for transparency and reproducibility. The top 15 upregulated and top 15 downregulated genes were selected based on fold change values and *p*-values < 0.05 for visualization in the heatmap (Figure 2E). KEGG pathway enrichment analysis of the differentially expressed genes revealed significant enrichment in stress-related and survival pathways, such as MAPK signaling, PI3K-Akt signaling, and apoptosis, as well as pathways associated with cellular fate decisions, including cell cycle and cellular senescence. Gene Ontology (GO) enrichment analysis further indicated that the differentially expressed genes were strongly associated with reproductive processes, such as reproductive structure development, female gonad development, and ovarian development. The volcano plot (Figure 2B) visualized the differential expression of genes, with the top 10 upregulated and top 10 downregulated genes highlighted based on fold change and *p*-value. Together, these results suggest a clear transcriptomic shift between control and heat-stressed ovaries.

### 2.3. Proteomic Alterations in Ovarian Tissue After Chronic Heat Stress

Proteomic profiling was conducted to analyze protein-level changes in ovarian tissues from control (CK) and heat-stressed (HS) mice following 30 days of chronic heat stress at 42 °C for 20 min daily (Figure 3). The initial dataset included a total of 9135 proteins, and after filtering, 8890 proteins were retained for further analysis. Differential expression analysis, using a fold change (|FC| ≥ 1.5) and *p*-value < 0.05 threshold, identified 59 upregulated and 116 downregulated proteins, while proteins not meeting these thresholds were classified as non-significantly different (Figure 3A). The full list of differentially expressed proteins (DEPs), along with their fold change and *p*-values, is provided in Supplementary Table S2 for transparency and reproducibility. Subcellular localization analysis revealed that the differentially expressed proteins were predominantly distributed in the nucleus (27.27%), extracellular region (14.88%), and mitochondria (9.92%) (Figure 3B). These findings suggest that heat stress influences nuclear regulation, extracellular signaling, and mitochondrial function in the ovary. KEGG pathway enrichment analysis of the differential proteins highlighted significant enrichment in pathways related to lipid metabolism, steroid hormone biosynthesis, and endocrine regulation, including cholesterol metabolism, PPAR signaling, Wnt signaling, and steroid hormone biosynthesis (Figure 3C). These pathways were linked to key biological functions involved in reproductive health and ovarian function under stress conditions. Gene Ontology (GO) functional enrichment analysis revealed that DEPs were strongly associated with metabolic processes, such as carboxylic acid biosynthesis, organic acid biosynthesis, and fatty acid biosynthetic processes (Figure 3D), highlighting the remodeling of ovarian metabolic pathways under heat stress. A heatmap of the top 15 upregulated and 15 downregulated proteins was generated (Figure 3E), showing distinct expression patterns between the control and heat-stressed groups. The heatmap was constructed based on fold change (FC ≥ 1.5) and adjusted *p*-value < 0.05, with purple representing upregulated proteins and teal representing downregulated proteins, indicating the relative expression levels across samples. The volcano plot (Figure 3A) visualized the differential expression of proteins, highlighting the top 10 upregulated and top 10 downregulated proteins based on fold change and *p*-value, supporting a clear proteomic shift between control and heat-stressed ovaries.

### 2.4. Metabolomic Alterations in Ovarian Tissue After Chronic Heat Stress

Untargeted metabolomic profiling was performed on ovarian tissues from control (CK) and heat-stressed (HS) mice following 30 days of chronic heat stress at 42 °C for 20 min daily, yielding a total of 3402 metabolites in both the raw and filtered datasets. Differential expression analysis, using a fold change (|FC| ≥ 1.5) and *p*-value < 0.05 threshold, identified 76 upregulated and 21 downregulated metabolites (Figure 4). The PLS-DA score plot (Figure 4A) showed a clear separation between CK and HS groups, with PLS-DA1 explaining 23.6% and PLS-DA2 explaining 12.2% of the total variance, indicating distinct metabolic shifts associated with chronic heat stress. The volcano plot (Figure 4B) visualized differential metabolite expression, highlighting the top 10 upregulated and top 10 downregulated metabolites based on fold change and *p*-value. Purple represents upregulated metabolites, and teal represents downregulated metabolites, supporting distinct metabolic alterations in response to heat stress. KEGG pathway enrichment analysis revealed significant enrichment in several metabolic pathways, including the TNF signaling pathway, cholesterol metabolism, PPAR signaling, and steroid hormone biosynthesis (Figure 4C). These findings suggest that heat stress induces metabolic reprogramming in the ovary, influencing key pathways involved in lipid and steroid metabolism. A heatmap of the top 15 upregulated and top 15 downregulated metabolites was generated (Figure 4D). The heatmap was constructed based on fold change (FC ≥ 1.5) and adjusted *p*-value < 0.05, with purple representing upregulated metabolites and teal representing downregulated metabolites. Functional annotations for these metabolites, based on biological pathways, are provided in Supplementary Table S3.

### 2.5. Integrated Analysis of Transcriptome, Proteome, and Metabolome Data

KEGG pathway and Gene Ontology (GO) enrichment analyses were performed using transcriptomic and proteomic datasets to identify biological processes and pathways affected by chronic heat stress. The enriched KEGG pathways included Wnt signaling, PPAR signaling, steroid hormone biosynthesis, cholesterol metabolism, and cytoskeleton-related pathways (Figure 5A). GO enrichment further indicated alterations in lipid transport, hormone secretion, ovulation cycle, and sex differentiation. These findings suggest that chronic heat stress is associated with changes in metabolic and reproductive regulatory pathways. Figure 5B presents a Venn diagram illustrating overlapping pathways among the transcriptomic, proteomic, and metabolomic datasets. A total of 16 overlapping pathways were identified, among which PPAR signaling, cholesterol metabolism, and steroid hormone biosynthesis were shared across all three omics layers (Figure 5C). These recurrently enriched pathways suggest that lipid metabolism and endocrine regulation may be central components of the ovarian response to chronic heat stress.

To further explore relationships among differentially expressed genes (DEGs), differentially expressed proteins (DEPs), and differential metabolites (DEMs), Spearman correlation analysis was performed. Gene-protein, gene-metabolite, and protein–metabolite correlations were evaluated, and the top 50 significant correlations were selected for visualization. The correlation network (Figure 6) shows molecular relationships across the three omics layers, with blue nodes representing genes, orange nodes representing proteins, and green nodes representing metabolites. Edges indicate significant correlations, with edge color representing the direction of correlation. Key interactions were observed among molecules associated with lipid metabolism, steroidogenesis, and reproductive regulation, supporting the integrated view that chronic heat stress affects interconnected molecular networks in ovarian tissue. The full correlation dataset, including correlation coefficients, *p*-values, and false discovery rates (FDRs), is provided in the Supplementary Files S4–S6.

### 2.6. RT-qPCR Validation of Selected Pathway-Related Genes

RT-qPCR was performed to validate the expression of selected genes involved in the key pathways identified through transcriptomic and proteomic analyses, specifically the PPAR signaling pathway, cholesterol metabolism, and steroid hormone biosynthesis. Genes were selected for validation based on their significant involvement in these pathways, as indicated by enrichment analyses and their representative roles in lipid metabolism and steroidogenesis. The selected genes included *Apoa2*, *Apoa1*, *Apoc1*, *Hsd17b2*, *Hsd3b6*, *Fads2*, *Lpl*, and *Lipg*, chosen for their pathway relevance, fold change in expression, and statistical significance in the omics data.

Consistent with the transcriptomic and proteomic results, heat stress (HS) significantly increased the mRNA expression levels of *Apoa2*, *Apoa1*, and *Apoc1*, which are involved in cholesterol metabolism and lipid transport, in comparison with the control group (Figure 7). In addition, *Hsd17b2*, *Hsd3b6*, and *Fads2*, key genes involved in steroid hormone biosynthesis, showed a marked upregulation under HS conditions, supporting altered regulation of steroidogenic pathways under heat stress (Figure 7). These findings corroborate the integrated omics data, supporting the involvement of lipid handling and steroid-related processes in the ovarian response to heat stress.

## 3. Discussion

Heat stress is one of the key environmental issues that may cause serious effects on the reproductive physiology of species. The increasing global temperatures and the fact that the frequency of heat waves has been on the increase have brought serious concerns as to the effect of thermal stress on the reproductive health of both animals and humans [30,31,32]. The ovary is highly vulnerable to environmental stress, as follicular growth, oocyte development, and steroid hormone synthesis are highly regulated metabolic and endocrine activities [33,34]. To investigate the molecular responses of mouse ovarian tissue to chronic heat stress in the current study, an integrated multi-omics approach combining transcriptomics, proteomics, and metabolomics was applied. The results of the current study indicate that the 30-day repeated daily heat-stress regimen was associated with molecular changes involving lipid metabolism, cholesterol metabolism, and steroid hormone synthesis. Histologically, the most consistent quantitative alteration was a reduction in secondary follicle profiles, whereas antral follicle profiles showed substantial inter-animal variability. These findings provide additional insights into how heat stress interferes with ovarian physiology.

Histological analysis showed that the number of secondary follicle profiles was significantly lower in the HS group, whereas primordial and primary follicle profiles were comparable between the groups. Antral follicle profiles showed inter-animal variability but remained comparable between groups. During histological evaluation, some follicles displayed degenerative morphological features, including disruption or partial detachment of the granulosa cell layers, pyknotic nuclei, oocyte degeneration, and partial collapse of the follicular structure. However, these features were not quantitatively classified as atretic follicles because some degenerative characteristics may overlap with normal follicular remodeling and antral follicle development. The dysfunction of granulosa cells and steroidogenesis is traditionally connected with follicular atresia [35]. The granulosa cells are critical in assisting the development of oocytes and the control of hormone levels in the ovarian follicles [36,37]. Therefore, the observed reduction in secondary follicle profiles and the variable occurrence of degenerative features may reflect altered follicular development under repeated heat exposure. These section-level morphological observations occurred alongside molecular alterations in lipid metabolism and steroidogenic pathways identified through the multi-omics analyses [38,39].

Transcriptomic findings also indicated that heat stress has a great impact on the expression of the genes relating to lipid metabolism as well as steroidogenic processes. Specifically, some lipid transport and fatty acid metabolism genes such as *Apoa1*, *Apoa2*, *Lpl* and *Fads2* were differentially expressed in the heat-stressed ovaries. These genes have significant functions in lipid transportation, metabolism of fatty acids, and homeostasis of cellular energy. Apoa1 and Apoa2 are the key protein constituents of the high-density lipoproteins that facilitate the transport of cholesterol and the maintenance of lipid homeostasis [40]. *Lpl* controls the absorption of lipids and triglyceride metabolism and plays a crucial role in the maintenance of lipid balance in metabolically active tissues [41]. *Fads2* is involved in polyunsaturated fatty acid biosynthesis that is vital to membrane structure and cellular signaling [42]. The change in expression of these genes indicates that heat stress can disrupt lipid metabolic pathways that are needed to sustain the metabolic environment needed to support follicular growth and oocyte maturation. Earlier research has indicated that lipid metabolism disruptions may affect granulosa cell activity, leading to oocyte developmental incompetence [43,44].

The transcriptomic results were supported by proteomic analysis, which revealed differences in the abundance of proteins related to metabolic and lipid transport. The alterations in protein expression associated with lipid metabolism suggest that not only is transcriptional control influenced by heat stress, but also the downstream molecular networks that maintain ovarian metabolic homeostasis. Since proteins are the main functional mediators of cellular activity, changes in protein levels can cause a direct effect on follicular growth and steroid hormone production. Proteomic analyses have revealed that environmental stress can cause major alterations in proteins that participate in metabolic processes and reproductive processes [45,46]. The consistency between transcriptomic and proteomic changes in this study implies that heat stress causes integrated molecular effects that interfere with major metabolic pathways in ovarian tissue.

Metabolomic profiling also showed that there were significant changes in the metabolites involved in lipid metabolism and steroid hormone biosynthesis pathways. Lipid-derived metabolites play a critical role in membrane composition, intracellular signaling, and energy metabolism, and are all vital in the development of the follicles and oocyte maturation [47]. The changes in steroid-associated metabolites indicate the possibility of heat stress disrupting the endocrine regulation in ovarian follicles too. The reproductive tissues of mice experiencing environmental stress have been reported to undergo similar metabolic responses in which metabolic remodeling influences the production of hormones and cellular levels of energy [25,27].

Transcriptomic, proteomic, and metabolomic data integration has found a few metabolic pathways that were highly enriched in various omics layers. Among them, the pathways associated with lipid metabolism, cholesterol metabolism, and steroid hormone production became one of the main factors in the ovarian response to prolonged heat stress. Steroid hormones are synthesized using cholesterol as the basic component, and it is necessary to regulate the transport and metabolism of cholesterol to ensure proper endocrine functioning in the ovary [27]. Loss of integrity in cholesterol metabolism may consequently degrade the production of estrogen and progesterone, which are major regulators of follicular development and reproductive cyclicity [48].

The integration of multi-omics also pointed to another pathway, namely the PPAR signaling pathway, which is a key pathway involved in maintaining lipid metabolism and cellular energy homeostasis. The peroxisome proliferator-activated receptors are transcription factors that influence genes of fatty acid metabolism, lipid transportation, and metabolic adjustment [49,50]. PPAR signaling in ovarian tissue plays a role in differentiating granulosa cells, developing follicles, and steroidogenesis [42,51]. The recurrent enrichment of PPAR signaling-related features across the integrated datasets suggests that repeated heat exposure may alter ovarian metabolic regulation through changes in lipid-responsive transcriptional networks.

The RT-qPCR validation provided different patterns of several key genes that were identified through the transcriptomic analysis, such as *Apoa1*, *Apoa2*, *Lpl*, *Fads2*, and *Hsd17b2*. Validation of these genes increases the reliability of transcriptomic findings and supports the role of lipid metabolism and steroid hormone regulation in the ovarian response to heat stress. Specifically, *Hsd17b2* is significant in steroid hormone metabolism as it controls the conversion of active and inactive steroid hormones [52]. Altered expression of this enzyme may influence estrogen metabolism and endocrine homeostasis in ovarian follicles.

Despite these findings, several limitations should be considered. First, no functional experiments were performed to specifically confirm the roles of the genes, proteins, metabolites, or pathways identified in this study. Future studies using targeted molecular and functional approaches will therefore be required to establish causal relationships between these molecular alterations and ovarian dysfunction. Second, the number of biological replicates was relatively limited across the analytical platforms, which may have reduced statistical power and contributed to inter-animal variability.

The estrous cycles of the mice were neither synchronized nor determined by vaginal cytology before ovarian tissue collection. Because ovarian cellular composition, follicular development, metabolic activity, and the relative synthesis of estrogens and progestogens vary across the estrous cycle, unequal representation of estrous stages may have influenced the histological observations and the transcriptomic, proteomic, and metabolomic profiles, particularly those related to steroidogenesis and ovarian metabolism. The available samples did not permit retrospective stratification or statistical adjustment according to estrous-cycle stage. Future studies should therefore include vaginal-cytology-based staging and balanced tissue collection across defined estrous stages.

The histological follicle counts were obtained from complete ovarian cross-sections but were not normalized to total section area, cortical area, or the cortex-to-stroma ratio and were not generated using an unbiased stereological approach. In the present study, one complete ovarian cross-section obtained during the original histological preparation from each ovary was evaluated for follicle-profile assessment. Although both ovaries from each mouse were independently examined and the counts from Ovary 1 and Ovary 2 were averaged to generate one animal-level value, multiple serial or non-consecutive sections throughout each ovary were not available for analysis. Therefore, the current approach provides section-level follicle-profile observations rather than a comprehensive stereological estimation of total ovarian follicular number. Differences in sectioning plane and cortical representation may therefore have influenced the absolute follicle-profile counts. Accordingly, these data should be interpreted as section-level follicle-profile observations rather than estimates of follicle density or the total ovarian follicular reserve. Separate biological cohorts were also used for histological, metabolomic, and matched transcriptomic–proteomic analyses because of the limited amount of ovarian tissue available from each mouse. Therefore, direct one-to-one comparison of the histological and molecular findings within the same animals was not possible.

All omics analyses were performed using whole ovarian tissue rather than separately isolated granulosa, theca, cumulus, or oocyte populations. Consequently, the identified molecular alterations represent composite whole-ovary responses and cannot be assigned to a specific ovarian cell type. Although the repeated heat-stress regimen was established based on previously reported protocols, additional physiological and molecular indicators of systemic heat stress, including rectal temperature measurements, serum corticosterone levels, and Hsp70 expression, were not evaluated in the present study. Additional functional markers related to apoptosis, oxidative stress, cellular proliferation, and steroidogenic activity were also not evaluated. Although transcriptomic findings were validated by RT-qPCR analysis, independent experimental validation at the protein level, such as Western blotting or immunohistochemistry, was not performed. Finally, since this research was conducted using a mouse model, caution should be taken when generalizing these results to other species. Future investigations incorporating larger cohorts, estrous-cycle staging, cell-type-specific analyses, systemic heat-stress indicators, functional experiments, and targeted protein-level validation would further strengthen the interpretation of the integrated multi-omics findings.

## 4. Materials and Methods

### 4.1. Animals and Ethics Statement

A total of 24 female C57BL/6 mice (8–12 weeks old, 22–26 g) were acquired from Chengdu Dasuo Experimental Animal Co., Ltd. (Chengdu, China). Mice were randomly assigned to two groups: control (CK) and heat-stressed (HS), with 12 mice per group. All animal procedures were performed in compliance with the experimental animal care and use guidelines of Sichuan Agricultural University (Sichuan, China) and were approved by the Experimental Animal Ethics Committee of Sichuan Agricultural University (approval no. 20250670).

### 4.2. Heat Stress Treatment

Mice were maintained in standard laboratory conditions in a 12 h light/dark cycle at a constant temperature of 21–22 °C and a relative humidity of 50–70%. They were fed standard chow and water ad libitum. Heat exposure was performed in a temperature-controlled environmental chamber. Mice in the HS group were exposed to 42 °C for 20 min once daily for 30 consecutive days, whereas CK mice were maintained under standard laboratory conditions. Each 20 min session represented a brief heat-exposure episode, and the overall experimental regimen is therefore referred to as repeated daily heat stress rather than continuous chronic heat exposure. During each heat exposure, animals were monitored for general condition and signs of distress. After each exposure session, mice were returned to standard housing conditions.

The repeated daily heat-stress regimen (42 °C for 20 min/day for 30 consecutive days) was established based on previously reported heat-stress protocols used in reproductive physiology studies [53]. Animals were monitored daily for general physiological condition throughout the exposure period.

### 4.3. Tissue Collection and Experimental Design

A total of 24 female C57BL/6 mice were used in this study, including 12 control (CK) mice and 12 heat-stressed (HS) mice. At the end of the 30-day repeated daily heat stress regimen, ovaries were collected for histological and multi-omics analyses. For all omics analyses, whole ovarian tissue was used rather than isolated ovarian cell populations. Granulosa cells, theca cells, cumulus cells, and cumulus-oocyte complexes were not separately isolated; therefore, each omics sample represented a mixed-cell whole-ovarian-tissue preparation.

For transcriptomic and proteomic analyses, whole ovarian tissue from six individual mice was analyzed, comprising three CK and three HS biological replicates. The same six mice were used for RNA-seq and DIA-based proteomic profiling to facilitate matched transcriptome-proteome comparisons and reduce inter-animal variability. RT-qPCR validation was performed using aliquots of the same RNA preparations used for RNA-seq. Thus, transcriptomic, proteomic, and RT-qPCR analyses each included three independent biological replicates per group.

For untargeted metabolomic profiling, whole ovarian tissue from ten individual mice was analyzed, comprising five CK and five HS biological replicates. A larger number of biological replicates was used for metabolomic analysis because metabolite abundance may be influenced by biological and technical variability, and additional replicates improve the robustness of metabolite detection and statistical comparison.

For histopathological analysis, both ovaries from eight individual mice, comprising four CK and four HS biological replicates, were fixed, paraffin-embedded, sectioned, and subjected to hematoxylin and eosin staining. One complete ovarian cross-section from each ovary obtained during the original histological preparation was evaluated for follicle-profile assessment in each mouse. Complete ovarian cross-sections from both ovaries of each mouse were examined. All identifiable follicle profiles within the entire available ovarian cross-section were counted according to morphological criteria rather than within selected microscopic fields. Follicle counts from Ovary 1 and Ovary 2 were averaged to generate one animal-level value, and each mouse was considered one independent biological replicate.

Because individual mouse ovaries provide limited tissue mass for simultaneous transcriptomic, proteomic, metabolomic, and histological analyses, separate biological cohorts were used for the different analytical platforms to ensure sufficient sample quantity and analytical reliability for each platform. Consequently, histological findings were used as supportive morphological evidence rather than direct one-to-one validation of the omics results from the same individual ovaries. An overview of the tissue type, sample allocation, and number of biological replicates used for each analytical platform is provided in Supplementary Table S7.

### 4.4. Ovarian Tissue Processing and Hematoxylin and Eosin Staining

Ovarian morphology was evaluated by hematoxylin and eosin (HE) staining after the 30-day repeated daily heat-stress regimen of 42 °C for 20 min once daily. Ovaries from four control (CK) and four heat-stressed (HS) mice were collected. Both the left and right ovaries of each mouse were stained with hematoxylin and eosin. Ovaries were fixed in 4% paraformaldehyde at room temperature, then dehydrated in a graded ethanol series, cleared in xylene, and embedded in paraffin for tissue processing. Paraffin blocks were cut serially with a microtome at a thickness of 4-5 μm. One complete ovarian cross-section from each ovary obtained during the original histological preparation was evaluated for follicle-profile analysis. The sections were deparaffinized in xylene, rehydrated in descending ethanol grades to water, and stained with hematoxylin. The sections were then rinsed, if needed, differentiated, blued, and counterstained with eosin. Slides were then processed for dehydration in a series of ethanol, clearing in xylene, and mounting in a resin-based mounting medium [54].

Stained sections were examined under bright-field microscopy under consistent imaging conditions across all groups. One complete ovarian cross-section from each ovary was examined rather than selected microscopic fields. All identifiable follicle profiles within the entire available cross-section were evaluated according to morphological criteria. Low-magnification images were used to show the complete ovarian cross-sections used for follicle-profile quantification, and corresponding higher-magnification regions were used only to illustrate the morphological criteria used for follicle identification and classification. Histological assessment focused on ovarian architecture and follicular integrity, including follicular development, follicle classification, granulosa cell layer organization, and general morphological features associated with tissue integrity [55].

Based on well-defined morphological criteria, the follicles were categorized as primary, secondary, and antral. Primary follicles contained a single layer of cuboidal granulosa cells, whereas secondary follicles contained two or more granulosa cell layers without a visible antral cavity. Antral follicles were identified only when a clearly defined antral cavity was visible. Follicles with inadequate tissue preservation, an unsuitable sectioning plane, or insufficiently clear morphology were excluded from stage-specific classification.

For quantitative analysis, follicle profiles were counted separately within the complete available cross-section from Ovary 1 and Ovary 2 of each mouse. The counts obtained from the two ovaries were subsequently averaged to generate one animal-level value for each follicular category, and each mouse was considered one independent biological replicate (n = 4 per group). The counts were not normalized to total section area, cortical area, or the relative proportions of ovarian cortex and stroma. Accordingly, the reported values represent follicle profiles observed in the analyzed complete ovarian cross-sections and should not be interpreted as follicle densities or stereological estimates of the total ovarian follicular reserve. Raw bilateral follicle-count data for individual biological replicates are provided in Supplementary Table S8.

### 4.5. Transcriptome Sequencing (RNA-Seq)

A standard Illumina-compatible mRNA library preparation protocol was used to prepare the RNA libraries with the service provider’s instructions (Novogene, China). The mRNA was denatured at high temperature, and first-strand cDNA was synthesized with random hexamer primers and M-MuLV (RNase H−) reverse transcriptase. All second-strand cDNA synthesis was done with DNA Polymerase I and RNase H. Following end repair and 3′ adenylation, hairpin-loop adapters were ligated to the fragments that were selected by size in the range of about 370–420 bp with AMPure XP beads. Universal and index primers were used to amplify libraries with Phusion High-Fidelity DNA polymerase. Libraries were quality-checked with the Agilent Bioanalyzer 2100 (Agilent Technologies, Santa Clara, CA, USA), and then normalization was performed prior to sequencing [56]. Sequencing was done on the NovaSeq platform (Illumina, San Diego, CA, USA) to achieve 150 bp paired-end reads using TruSeq PE Cluster Kit v3-cBot-HS (Illumina, San Diego, CA, USA), with clustering of index-coded samples [57].

Before the preparation of the libraries, the quality and integrity of the total RNA were evaluated on agarose gel electrophoresis, NanoDrop spectrophotometry, and Agilent 2100 Bioanalyzer. All RNA samples had OD260/280 ratios ranging from 1.8 to 2.0 and RNA integrity number (RIN) values greater than 7.0 for sequencing purposes. Prior to sequencing, the Agilent Bioanalyzer 2100 system was used to assess the quality of the libraries.

Sequencing was performed on the Illumina NovaSeq platform (150 bp paired-end reads). About 50 million paired-end reads per sample were sequenced. Adapters, poly-N reads, and low-quality reads were removed from the raw reads. Quality control metrics such as Q20 and Q30 scores were computed, Q20 should be above 95% and Q30 above 85%, to get good quality data to carry out further analysis. To align clean reads to the mouse reference genome, HISAT2 (v2.0.5) was used; for transcript assembly, StringTie (v1.3.3b) was used. Read counts at the gene level were obtained by assigning features with featureCounts (v1.5.0-p3), and expression was normalized as FPKM [58]. All samples were subjected to downstream differential expression analysis after filtering out transcripts that had very low read counts to minimize noise from lowly expressed genes.

The differentially expressed genes (DEGs) were obtained by using DESeq2 (v1.20.0) with Benjamini–Hochberg (BH) correction. Genes that exhibited a fold change of ≥1.5 or ≤0.67 with *p*-values adjusted at *p* < 0.05 were deemed to be significantly differentially expressed. ClusterProfiler package was used to perform functional enrichment analysis, including Kyoto Encyclopedia of Genes and Genomes (KEGG) and Gene Ontology (GO), with corrected *p*-values < 0.05 being significant. Gene set enrichment analysis was performed using the gene sets from GO and KEGG [59].

### 4.6. Proteome Analysis (DIA-Based LC–MS/MS)

The same biological samples (ovarian tissues) were used for proteomic profiling in 3 control (CK) and 3 heat-stressed (HS) mice after 30 days of heat exposure. Tissues were ground in liquid nitrogen and lysed in SDT buffer containing DTT, then ultrasonicated on ice, and centrifuged (12,000× *g*, 15 min, 4 °C). The supernatants were boiled, cooled, reacted with iodoacetamide in the dark, and protein-precipitated with pre-chilled acetone. The proteins were washed, air-dried, and redissolved in DB buffer (6 M urea, 100 mM TEAB, pH 8.5). The amount of protein was measured by Bradford assay, and SDS–PAGE was performed to analyze the quality of the protein after staining the gels with Coomassie Brilliant Blue. Proteins were digested with trypsin at 37 °C, and the digestion was stopped with formic acid (pH < 3), which was then desalted with C18 cartridges, eluted, and lyophilized [60].

Data-independent acquisition (DIA) mode was used to reconstruct peptides and analyze them with a Vanquish Neo UHPLC system and a Thermo Astral mass spectrometer. The DIA method used a C18 pre-column (5 μm × 300 μm, 5 μm, Thermo) and a C18 analytical column (150 μm × 15 cm, 2 μm, Thermo). The elution gradient for liquid chromatography was as follows: at 0 min, 96% mobile phase A and 4% mobile phase B; at 0.2 min, 95% A and 5% B; at 4 min, 75% A and 25% B; at 5.8 min, 65% A and 35% B; at 6.2 min, 1% A and 99% B; at 6.9 min, 1% A and 99% B, then stop run. The mass spectrometer was run in positive ion mode with an ESI source (Easy-spray). The ion spray voltage was adjusted to 2.0 kV, and the ion transfer tube temperature was adjusted to 290 °C [61].

The raw MS data were processed with the DIA-NN software for protein identification and quantification, with fixed modification of carbamidomethylation on cysteine and variable modification of N-terminal methionine. A maximum of 2 miscleavages were allowed. Global was used to filter out identifications. Q.Value < 0.01 (peptides) and PG.Q.Value < 0.01 (proteins). Statistical tests were performed to identify differentially expressed proteins, depending on the distribution of the data. Benjamini–Hochberg false discovery rate (FDR) correction was performed to control for multiple testing. The normality of protein abundance distributions was evaluated by the Shapiro–Wilk test. Comparisons between groups were conducted with Student’s *t*-test for data that were normally distributed and with Mann–Whitney U test for data that were not normally distributed. Proteins that had a fold change of >1.5 or <0.67 and *p*-values < 0.05 were deemed to be significantly altered [62].

### 4.7. Untargeted Metabolomic Profiling (UHPLC–MS/MS)

Untargeted metabolomic profiling was conducted using ovarian tissues collected after the 30-day heat exposure at 42 °C for 20 min daily (5 CK and 5 HS biological samples). For each metabolomic biological replicate, ovarian tissues collected from both ovaries of the same mouse were pooled before metabolite extraction because of the relatively small size of individual mouse ovaries. Tissue samples were homogenized in pre-chilled 80% methanol for metabolite extraction and subsequent UHPLC–MS/MS analysis [63].

A Vanquish UHPLC system interfaced with a high-resolution Orbitrap Q Exactive mass spectrometer was used for chromatographic separation. The injection volume was 10 μL, and the linear gradient was 12 min with a flow rate of 0.2 mL/min on a Hypersil Gold column (100 × 2.1 mm, 1.9 μm). The solvent gradient was set as follows: 2% B for 1.5 min, 2–85% B for 3 min, 85–100% B for 10 min, 100–2% B for 10.1 min, and 2% B for 12 min. The mass spectrometer was analyzed in positive ion and negative ion modes with a spray voltage of 3.5 kV, capillary temperature of 320 °C, sheath gas flow rate of 35 psi, and auxiliary gas flow rate of 10 L/min [64].

Quality control (QC) samples were prepared by pooling aliquots from each biological sample to ensure data quality. The QC sample was injected repeatedly during the analysis to control the stability of the system and signal drift. Signal drift correction was applied to compensate for signal drift and minimize possible batch effects by normalizing the metabolite peak intensities using the responses from the QC samples. MetaX software (version 1.0) was used to correct for batch effects, and the batch effect was minimized further [65].

Peak alignment, peak picking and quantification of metabolites were performed using software XCMS. Metabolite identification was carried out using adduct ions by matching with a mass deviation of 10 ppm with a self-constructed high-quality secondary spectrum database. Blank samples were used to remove background ions, and relative peak areas were normalized. Sample characteristics that showed a coefficient of variation greater than 30% in QC samples were not included in further analysis [66].

MetaX was used for multivariate analyses, such as principal component analysis (PCA) and partial least squares discriminant analysis (PLS-DA). PLS-DA model was validated by cross-validation and permutation testing for robustness and the risk of overfitting the model. The multiple testing correction was done using Benjamini–Hochberg FDR correction. All metabolites that showed a fold change ≥1.5 or ≤0.67 and an adjusted *p* < 0.05 were deemed to be significantly altered. These metabolites were mapped into KEGG pathways, and metabolites with pathways that were significantly enriched (corrected *p* < 0.05) were identified [67].

### 4.8. Functional Enrichment Analysis

The sets of differentially expressed genes (DEGs), differentially expressed proteins (DEPs), and differential metabolites (DEMs) between the control and heat-stressed groups were used for functional enrichment analyses in R software (version 4.5.0). The DEGs were converted to corresponding gene identifiers for transcriptomic enrichment, DEPs were mapped by UniProt identifiers for proteomic enrichment, and DEMs were annotated and mapped to KEGG compound identifiers for metabolomic enrichment. Only features with a very large fold change (at least >1.5 or <0.67) that satisfy the statistical thresholds in the differential screening step of each omics analysis were used as input. Based on pathway mapping with the Kyoto Encyclopedia of Genes and Genomes (KEGG), Gene Ontology (GO) and Kyoto Encyclopedia of Genes and Genomes (KEGG) enrichment analyses for the DEGs and DEPs were performed, while KEGG pathway enrichment was performed for the DEMs. Biological processes and signaling pathways related to chronic heat stress-induced ovarian injury were interpreted using enriched terms and pathways.

### 4.9. Real-Time Quantitative PCR Analysis

The protocol of RT-qPCR was followed as described by [68]. The ovarian tissue was harvested, and total RNA was extracted using the RNAiso reagent (Takara, Otsu, Japan), and cDNA was synthesized using the PrimeScript RT reagent Kit (ThermoFisher, Waltham, MA, USA) according to the manufacturer’s instructions. The ABI 7300 Real-Time PCR System (Applied Biosystems, Foster City, CA, USA) using SYBR Green chemistry (Bio-Rad, Hercules, CA, USA) was used for quantitative PCR. The reaction conditions were an initial denaturation step at 95 °C for 5 min, followed by 40 cycles of 95 °C for 10 s and 60 °C for 30 s. Triplicate tests were performed on each sample. The relative gene expression was standardized against β-actin and normalized by calculating the 2^−ΔΔCt^ value. Changes in gene expression were measured relative to the control group (non-heat-stressed mice). The primers for each target gene are listed in the primer table. Each primer pair was tested for its efficiency, and all the primers exhibited efficiencies ranging from 90 to 110. Software was used to confirm reference gene (β-actin) stability in all experimental conditions for reliable normalization of the data [69]. Primer sequences for the target genes are shown in Table 1.

### 4.10. Statistical Analysis

R software (version 4.3.2) and GraphPad Prism (version 10.0) were used for statistical analyses. Data are presented as mean ± standard deviation (SD). Data from RT-qPCR analysis were expressed as fold changes in gene expression relative to the reference gene, and differences between the gene expression levels of the CK and HS groups were analyzed by unpaired two-tailed Student’s *t*-test in GraphPad Prism. Statistical significance was defined as a *p*-value of <0.05. The subsections dealing with transcriptomic, proteomic, and metabolomic analyses provide detailed statistical methods and filters for the transcriptomic, proteomic, and metabolomic analyses, respectively.

## 5. Conclusions

This study demonstrated that the 30-day repeated daily heat stress induced coordinated transcriptomic, proteomic, and metabolic changes in mouse ovarian tissue, particularly in pathways associated with lipid metabolism, cholesterol transport, PPAR signaling, and steroid hormone biosynthesis. Histological examination revealed a significant difference in the number of secondary follicle profiles, while primary and antral follicle profiles showed inter-animal variability without significant differences between control and heat-stressed animals. Because degenerative morphological features may overlap with normal follicular remodeling, atretic follicle profiles were not quantitatively evaluated in the revised histological assessment. Integration of the molecular changes across the three omics layers suggests that interconnected regulatory pathways involving lipid metabolism and steroid biosynthesis are important components of the ovarian response to repeated heat exposure. The results provide a systems-level perspective on how heat stress affects ovarian function and suggest specific candidate molecules and pathways for further investigation. To determine causal relationships underlying these molecular changes, future studies involving larger cohorts, estrous-stage-controlled animals, cell-specific analyses, and targeted functional validation are needed.

## Figures and Tables

**Figure 1:**
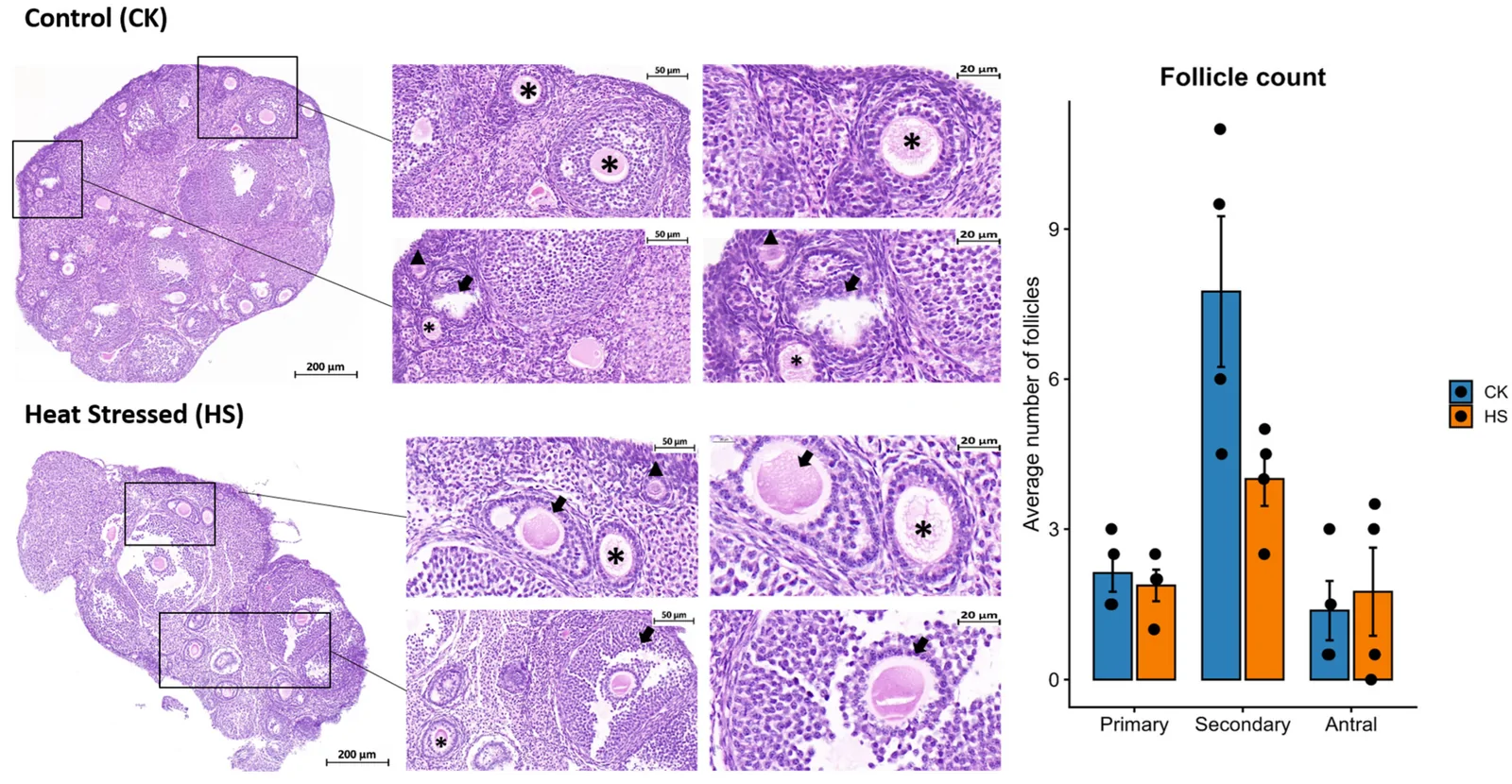
Histological evaluation and quantification of ovarian follicle profiles in control (CK) and heat-stressed (HS) mice. Representative hematoxylin and eosin (H&E)-stained complete ovarian cross-sections are shown for the HS group (top row) and the CK group (bottom row) at low magnification (scale bar = 200 µm). The boxed regions from the complete ovarian cross-sections are enlarged at higher magnifications (scale bars = 50 µm and 20 µm) to demonstrate follicle morphology and classification criteria. These higher-magnification images were used for morphological illustration only and were not considered independent counting fields. Black filled triangles (^▲^) indicate primary follicles, asterisks (*) indicate secondary follicles, and arrows indicate antral follicles. Follicles were classified according to predefined morphological criteria: primary follicles were identified as oocytes surrounded by a single layer of cuboidal granulosa cells; secondary follicles were identified as oocytes surrounded by two or more granulosa cell layers without a visible antral cavity; and antral follicles were identified only when a clearly visible antral cavity was present. For quantitative histological analysis, both ovaries from each mouse were examined, and follicle counts from Ovary 1 and Ovary 2 were averaged to generate one animal-level value for each follicular category. The bar graph shows the mean ± SD, and black dots represent individual biological replicates (*n* = 4 mice per group).

**Figure 2:**
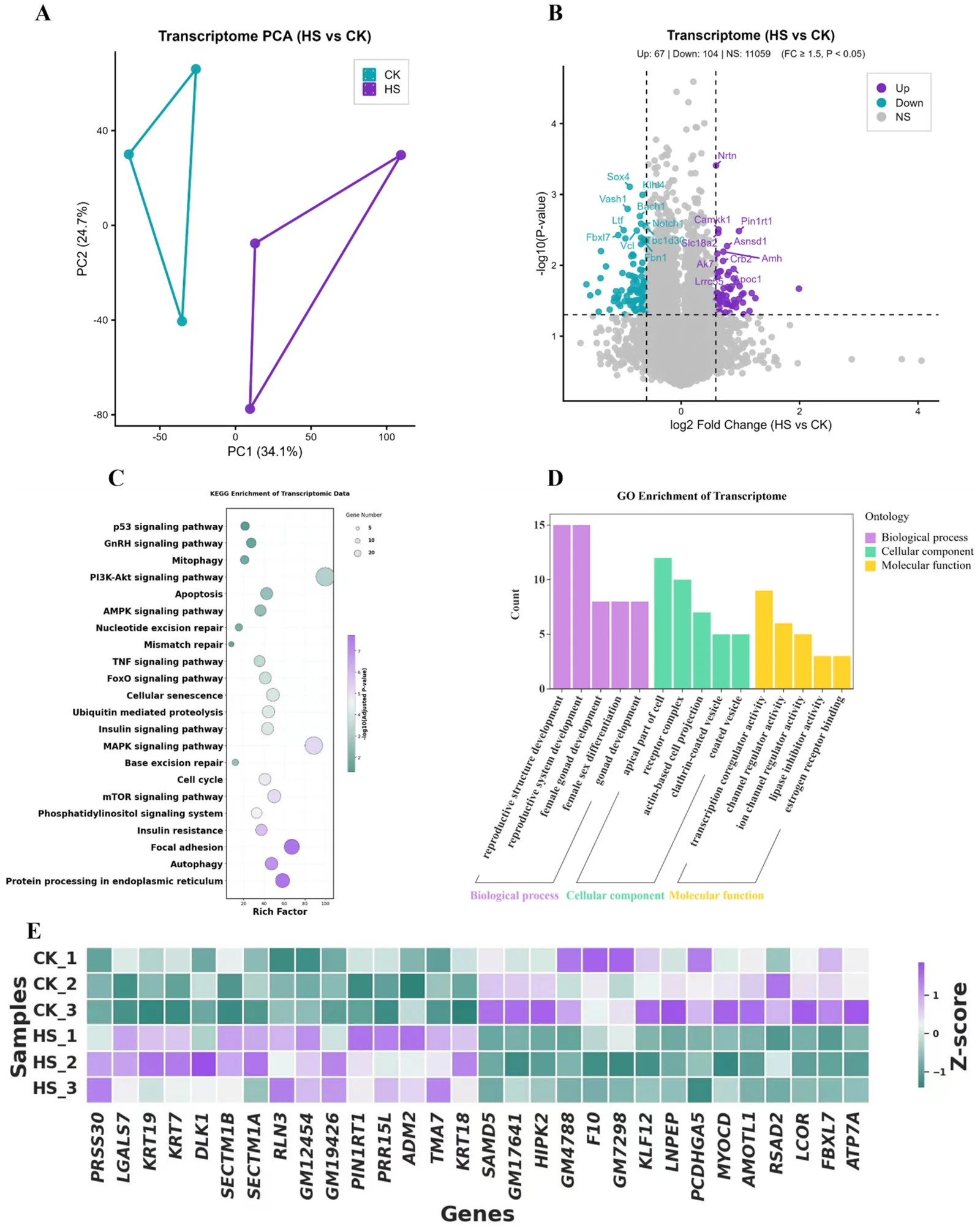
Transcriptomic analysis of ovarian tissues from control (CK) and heat-stressed (HS) mice. (**A**) PCA showing separation between CK and HS groups (PC1: 34.1%, PC2: 24.7%). (**B**) Volcano plot highlighting the top 10 upregulated and downregulated genes (|FC| ≥ 1.5, *p* < 0.05). (**C**) KEGG pathway enrichment analysis. (**D**) GO enrichment analysis for reproductive and ovarian processes. (**E**) Heatmap of the top 15 upregulated and downregulated genes, with purple for upregulated and teal for downregulated.

**Figure 3:**
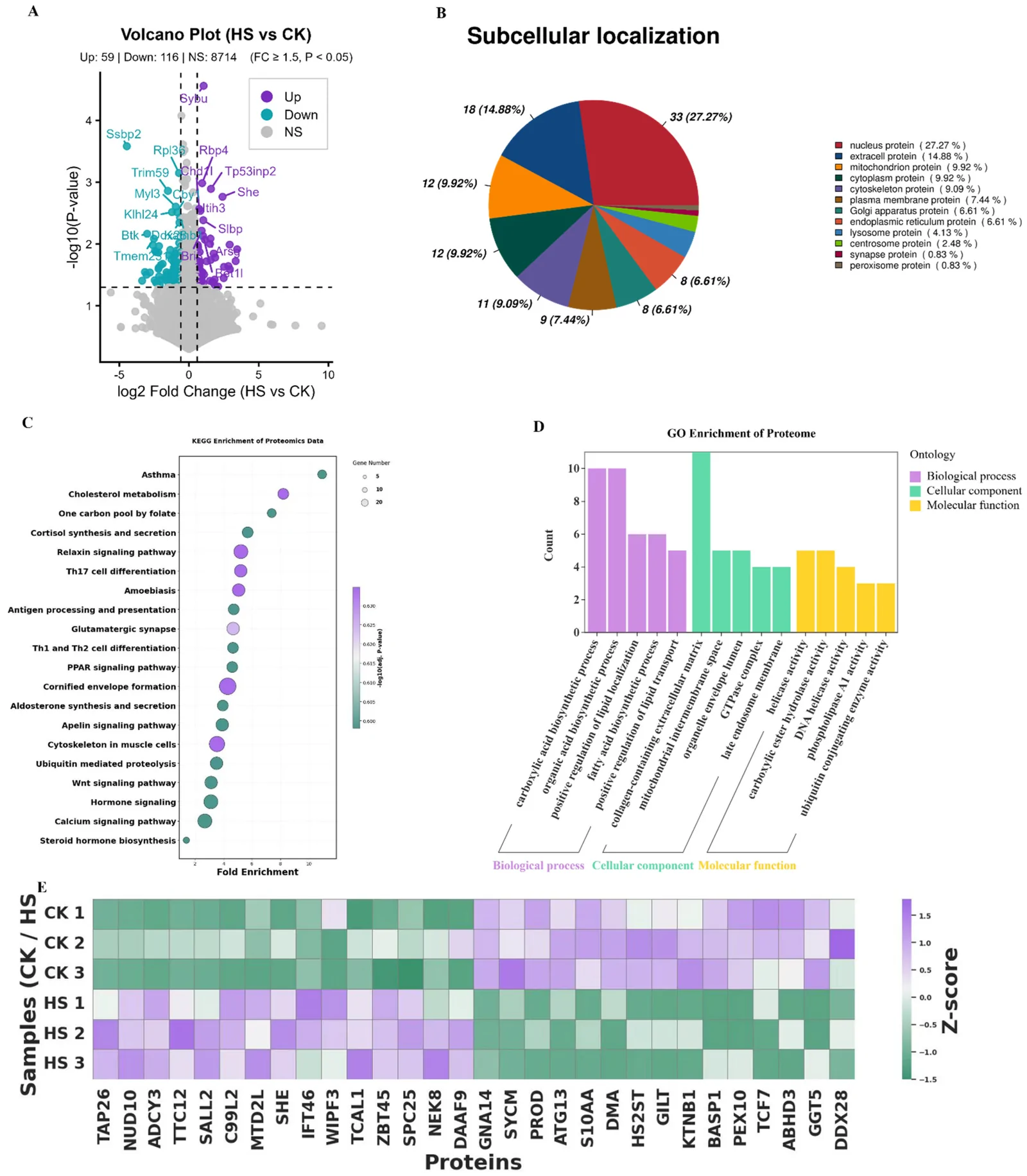
Proteomic analysis of ovarian tissues from control (CK) and heat-stressed (HS) mice. (**A**) Volcano plot of DEPs highlighting the top 10 upregulated and downregulated proteins (|FC| ≥ 1.5, *p* < 0.05). (**B**) Subcellular localization of DEPs. (**C**) KEGG pathway enrichment analysis. (**D**) GO enrichment analysis for metabolic processes. (**E**) Heatmap of the top 15 upregulated and downregulated proteins, with purple for upregulated and teal for downregulated.

**Figure 4:**
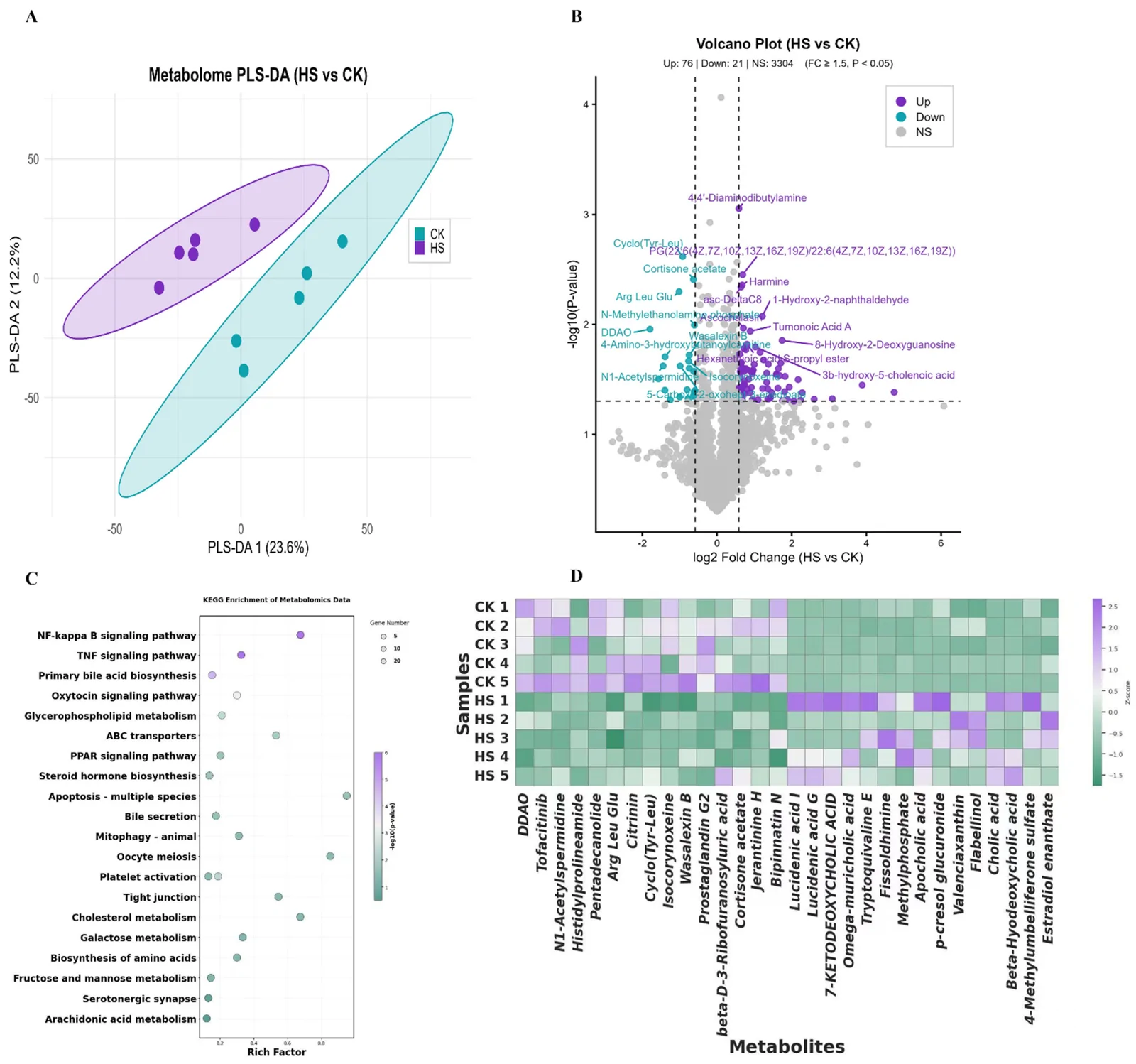
Metabolomic analysis of ovarian tissues from control (CK) and heat-stressed (HS) mice. (**A**) PLS-DA score plot showing separation between CK and HS groups. (**B**) Volcano plot highlighting the top 10 upregulated and downregulated metabolites (|FC| ≥ 1.5, *p* < 0.05). Purple represents upregulated metabolites, and teal represents downregulated metabolites. (**C**) KEGG pathway enrichment analysis of differentially expressed metabolites. (**D**) Heatmap of the top 15 upregulated and 15 downregulated metabolites (|FC| ≥ 1.5, *p* < 0.05), with purple for upregulated and teal for downregulated metabolites.

**Figure 5:**
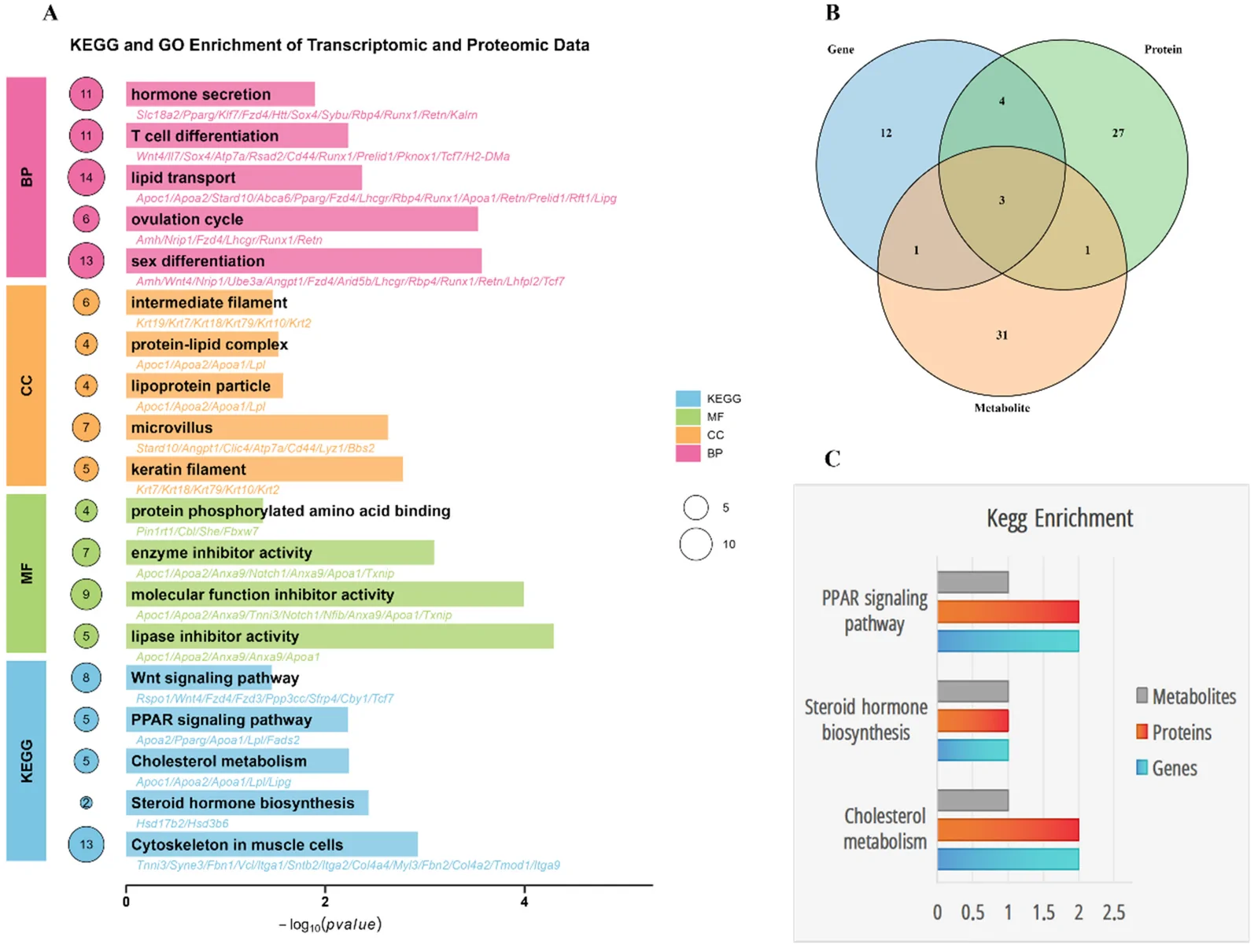
Integrated enrichment analysis of transcriptomic, proteomic, and metabolomic datasets. (**A**) KEGG and GO enrichment analysis of differentially expressed genes and proteins. (**B**) Venn diagram showing overlapping pathways across transcriptomic, proteomic, and metabolomic datasets. (**C**) Shared enriched pathways across all three omics layers, including PPAR signaling, cholesterol metabolism, and steroid hormone biosynthesis.

**Figure 6:**
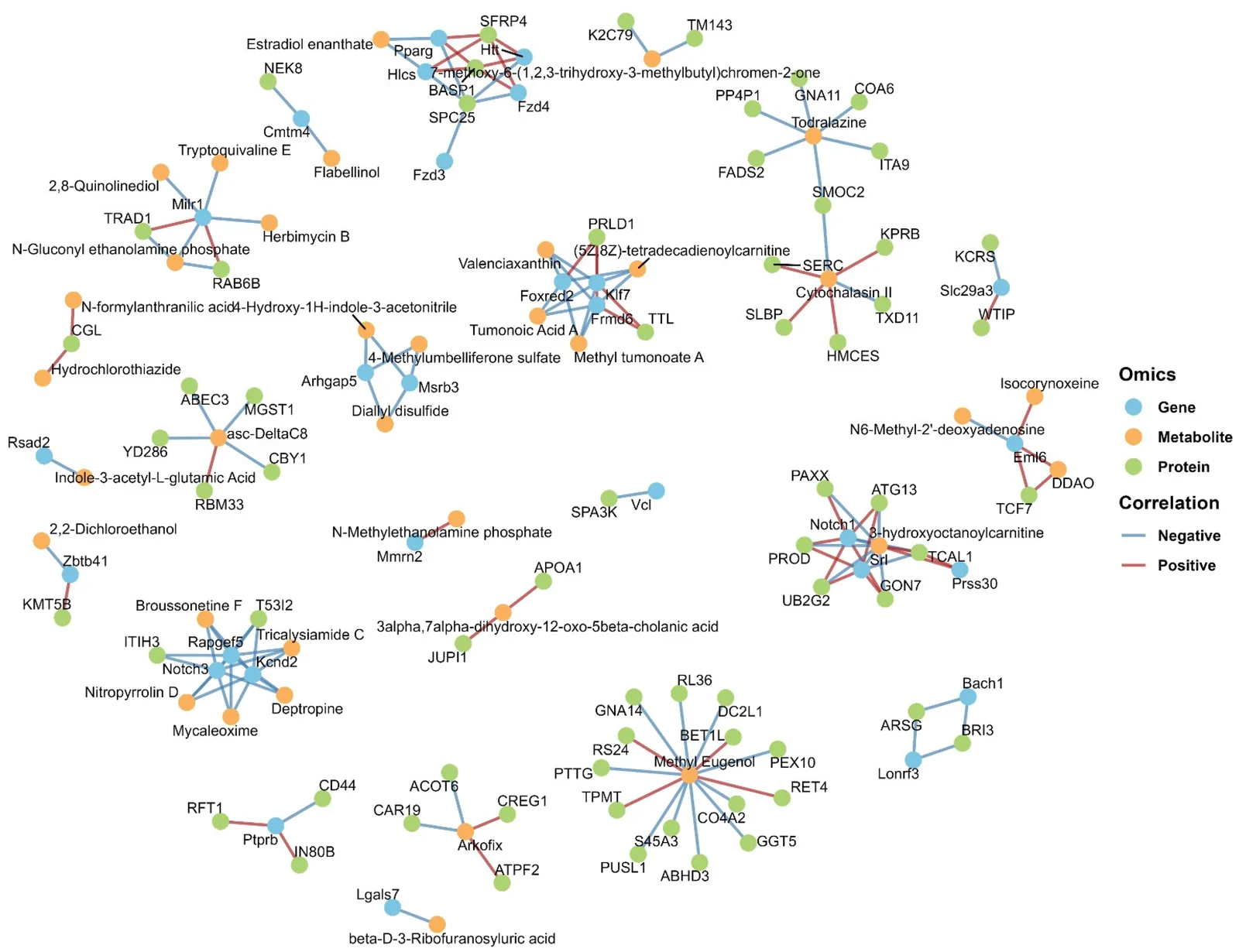
Correlation network of the top 50 gene, protein, and metabolite associations. Nodes represent genes, proteins, and metabolites, shown in blue, orange, and green, respectively. Edges represent significant correlations among molecular features across transcriptomic, proteomic, and metabolomic datasets.

**Figure 7:**
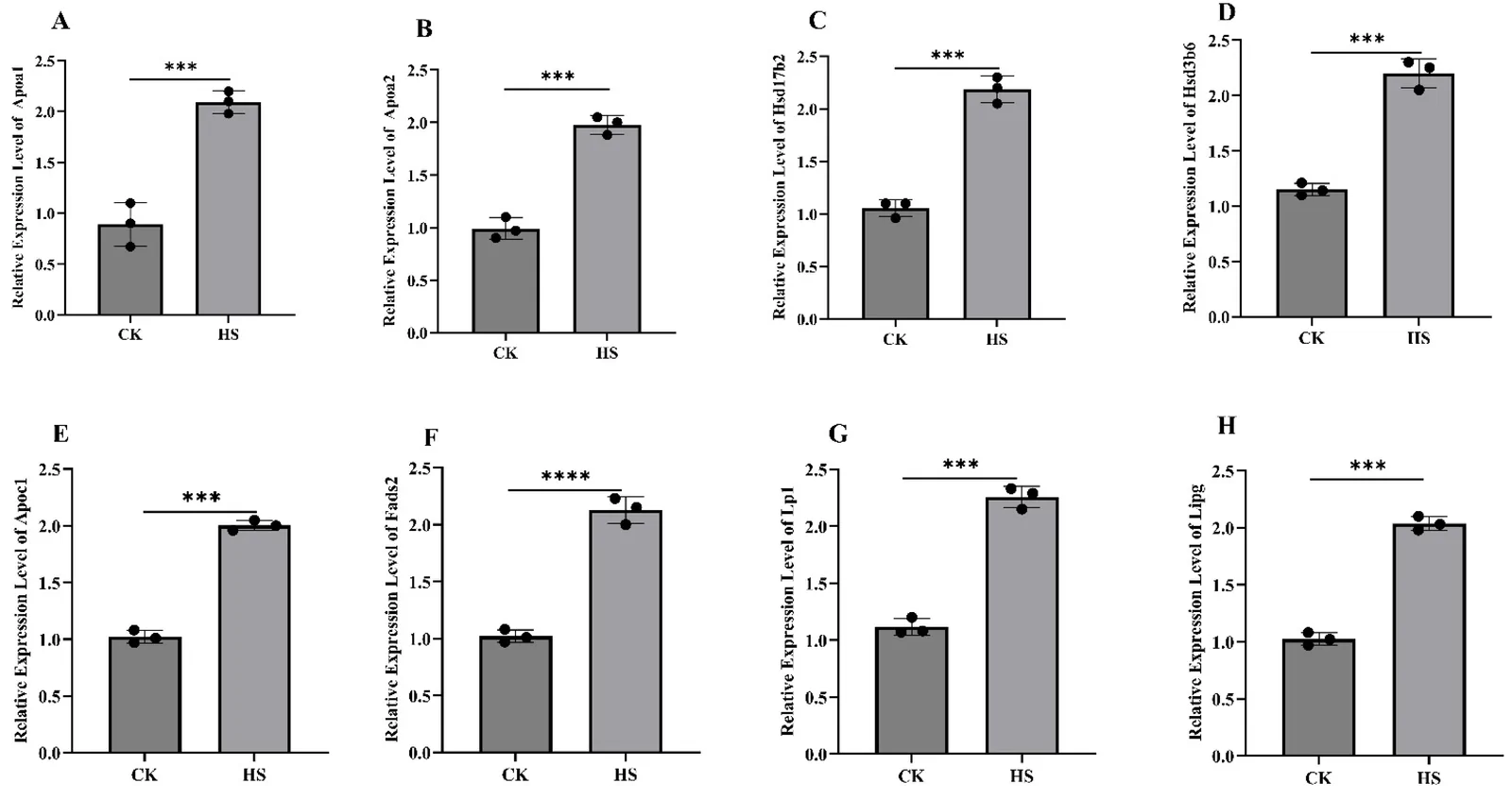
RT-qPCR validation of pathway-related candidate genes derived from transcriptomic and proteomic analyses. Relative mRNA expression levels of selected genes in ovarian tissues from CK and HS groups are shown. (**A**) *Apoa1* expression level; (**B**) *Apoa2* expression level; (**C**) *Hsd17b2* expression level; (**D**) *Hsd3b6* expression level; (**E**) *Apoc1* expression level; (**F**) *Fads2* expression level; (**G**) *Lpl* expression level; (**H**) *Lipg* expression level. These targets were selected as representative genes from enriched pathways (PPAR signaling pathway, cholesterol metabolism, and steroid hormone biosynthesis) identified across omics layers. Data are presented as mean ± SD with individual biological replicates shown as dots (*n* = 3 per group). Statistical differences between the CK and HS groups were evaluated using an unpaired two-tailed Student’s *t*-test. *** *p* < 0.001, and **** *p* < 0.0001.

**Table 1:** Primer sequences used for RT-qPCR.

Gene	Gene ID	Primer Direction	Primer Sequence (5′–3′)	Product Size (bp)
*Apoa1*	NM_009692.4	Forward	GAACGAGTACCACACCAGGG	91
		Reverse	CATCAGACTATGGCGCAGGT	
*Apoa2*	NM_001305550.1	Forward	ATAGCCCCTACCTCCAGTCA	72
		Reverse	TGGCAGACTATGGCCGATTC	
*Apoc1*	NM_001110009.2	Forward	GAGAGATCCTTAGATCCAGGGTG	357
		Reverse	ACCTGGCACATTACGTGGAT	
*Fads2*	NM_019699.2	Forward	ACTGGCTGGATTCCTACCCT	79
		Reverse	AGTCATGTTGCAGCCATCCA	
*Hsd17b2*	NM_001424829.1	Forward	TTCGGAGACCGAGTCCTACT	81
		Reverse	CAGAACCAGTGCTCTGCTCA	
*Hsd3b6*	NM_013821.3	Forward	AACCTCAGAGTGGATCGGGA	120
		Reverse	TCCTTACTGGAGGACCTGGTA	
*Lipg*	NM_010720.3	Forward	CGGCCTGTCCACTTCTAGTCT	198
		Reverse	AGGTTGTGATACTTCCCGCC	
*Lpl*	NM_008509.2	Forward	TCGCCTTTCTCCTGATGACG	171
		Reverse	GCAATCACACGGATGGCTTC	

Note: All primers were amplified at a uniform annealing temperature of 60 °C. Although most RT-qPCR amplicons were shorter than 200 bp, the Apoc1 primer pair generated a 357 bp product. This primer pair was retained because it produced a single melting-curve peak under the optimized amplification conditions used in this study.

## Data Availability

The original contributions presented in this study are included in the article. Further inquiries can be directed to the corresponding authors.

## Supplementary Materials

The supporting information can be downloaded at https://www.mdpi.com/article/10.3390/ijms27198671/s1.

## Abbreviations

The following abbreviations are used in this manuscript:

ABI	Applied Biosystems
Akt	Protein kinase B
AMPure XP	AMPure XP magnetic beads
Apoa1	Apolipoprotein A1
Apoa2	Apolipoprotein A2
Apoc1	Apolipoprotein C1
bp	Base pair
C18	Octadecyl/reversed-phase chromatography material
C57BL/6	C57 black 6 mouse strain
CC BY	Creative Commons Attribution
cDNA	Complementary DNA
CK	Control
DB	Dissolution buffer
DEGs	Differentially expressed genes
DEMs	Differentially expressed metabolites
DEPs	Differentially expressed proteins
DESeq2	Differential expression analysis software for RNA-seq count data
DIA	Data-independent acquisition
DIA-NN	Data-independent acquisition neural network software
DNA	Deoxyribonucleic acid
DTT	Dithiothreitol
ESI	Electrospray ionization
FC	Fold change
FDR	False discovery rate
FPKM	Fragments per kilobase of transcript per million mapped reads
GO	Gene Ontology
HE	Hematoxylin and eosin
HF	High field
HISAT2	Hierarchical Indexing for Spliced Alignment of Transcripts 2
HS	Heat stress/heat-stressed
Hsd17b2	Hydroxysteroid 17-beta dehydrogenase 2
Hsd3b6	Hydroxy-delta-5-steroid dehydrogenase, 3 beta- and steroid delta-isomerase 6
Hsp70	Heat shock protein 70
KEGG	Kyoto Encyclopedia of Genes and Genomes
LC-MS/MS	Liquid chromatography–tandem mass spectrometry
Lipg	Endothelial lipase
Lpl	Lipoprotein lipase
MAPK	Mitogen-activated protein kinase
M-MuLV	Moloney murine leukemia virus
mRNA	Messenger RNA
MS	Mass spectrometry
OD260/280	Optical density ratio at 260 and 280 nm
PC1	Principal component 1
PC2	Principal component 2
PCA	Principal component analysis
PCR	Polymerase chain reaction
PE	Paired-end
PG.Q.Value	Protein group q-value
PI3K	Phosphoinositide 3-kinase
PLS-DA	Partial least squares discriminant analysis
PPAR	Peroxisome proliferator-activated receptor
QC	Quality control
qPCR	Quantitative polymerase chain reaction
RIN	RNA integrity number
RNA	Ribonucleic acid
RNA-seq	RNA sequencing
RNase	Ribonuclease
RT-qPCR	Reverse transcription quantitative polymerase chain reaction
SD	Standard deviation
SDT	SDS-DTT-Tris buffer
SDS-PAGE	Sodium dodecyl sulfate-polyacrylamide gel electrophoresis
SYBR	SYBR Green fluorescent DNA-binding dye
TEAB	Triethylammonium bicarbonate
TNF	Tumor necrosis factor
UHPLC	Ultra-high-performance liquid chromatography
UHPLC-MS/MS	Ultra-high-performance liquid chromatography–tandem mass spectrometry
Wnt	Wingless/Int signaling pathway
XCMS	Mass spectrometry data-processing software
