# Supplementary material for: Integrated Multi-Omics Reveals Disruption of Lipid–Steroid Regulatory Axis Underlying Chronic Heat Stress-Induced Ovarian Dysfunction in Mice
Source: Int J Mol Sci. 2026 Sep 28;27(19):8671. doi: 10.3390/ijms27198671 (PMC13664949; doi:10.3390/ijms27198671)
Supplement: Supplementary file 1 [file ijms-27-08671-s001.zip › Table S7.pdf]

Overview of tissue types and biological replicates used for each analysis

Analytical platform	Sample type	CK	HS	Total	Biological unit
RNA-seq	Whole ovarian tissue; no cell-type isolation	3	3	6	One mouse per replicate
DIA proteomics	Whole ovarian tissue from the same mice used for RNA-seq	3	3	6	One mouse per replicate
RT-qPCR	Aliquots of the same RNA preparations used for RNA-seq	3	3	6	One mouse per replicate
Untargeted metabolomics	Whole ovarian tissue; no cell-type isolation	5	5	10	One mouse per replicate
Histology	Both ovaries; complete ovarian cross-sections	4	4	8	One mouse per replicate
