# Supplementary material for: Integrated Multi-Omics Reveals Disruption of Lipid–Steroid Regulatory Axis Underlying Chronic Heat Stress-Induced Ovarian Dysfunction in Mice
Source: Int J Mol Sci. 2026 Sep 28;27(19):8671. doi: 10.3390/ijms27198671 (PMC13664949; doi:10.3390/ijms27198671)
Supplement: Supplementary file 1 [file ijms-27-08671-s001.zip › Table S8.pdf]

Individual animal-level follicle counts on bilateral ovarian cross-section analysis

Samples	Primary Follicle		Secondary Follicle		Antral Follicle	
	Ovary 1	Ovary 2	Ovary 1	Ovary 2	Ovary 1	Ovary 2
**CK 1**	1	2	11	8	3	3
**CK 2**	2	3	6	6	1	2
**CK 3**	4	2	12	10	0	1
**CK 4**	3	0	9	0	1	0
**HS 1**	4	0	5	3	4	2
**HS 2**	2	2	7	3	1	0
**HS 3**	3	2	5	4	0	0
**HS 4**	1	1	5	0	2	5

Average follicle count per mouse=(Number of follicles in Ovary 1 + Number of follicles in Ovar y 2 /) 2

Sample	Group	Primary follicle	Secondary follicle	Antral follicle
CK1	CK	1.5	9.5	3
CK2	CK	2.5	6	1.5
CK3	CK	3	11	0.5
CK4	CK	1.5	4.5	0.5
HS1	HS	2	4	3
HS2	HS	2	5	0.5
HS3	HS	2.5	4.5	0
HS4	HS	1	2.5	3.5

Group mean ± SD

Group	Primary follicle	Secondary follicle	Antral follicle
CK	2.13 ± 0.85	7.75 ± 2.99	1.38 ± 1.19
HS	1.88 ± 0.63	4.00 ± 1.08	1.75 ± 1.71
